# A simple method to determine changes in the affinity between HisF and HisH in the Imidazole Glycerol Phosphate Synthase heterodimer

**DOI:** 10.1371/journal.pone.0267536

**Published:** 2022-04-22

**Authors:** Vitor M. Almeida, J. Patrick Loria, Sandro R. Marana

**Affiliations:** 1 Departamento de Bioquímica, Instituto de Química, Universidade de São Paulo, São Paulo, São Paulo, Brazil; 2 Department of Chemistry, Yale University, New Haven, Connecticut, United States of America; 3 Department of Molecular Biophysics and Biochemistry, Yale University, New Haven, Connecticut, United States of America; Weizmann Institute of Science, ISRAEL

## Abstract

The bi-enzyme HisF-HisH heterodimer is part of the pathway that produces histidine and purines in bacteria and lower eukaryotes, but it is absent in mammals. This heterodimer has been largely studied probing the basis of the allosteric effects and the structural stability in proteins. It is also a potential target for antibacterial drugs. In this work, we developed a simple method to evaluate changes in the affinity between HisF and HisH in the heterodimer of the bacteria *Thermotoga maritima*. HisH contains a single tryptophan residue, which is exposed in the free protein, but buried in the heterodimer interface. Hence, the intrinsic fluorescence maximum of this residue changes to shorter wavelengths upon dimerization. Thus, we used the fluorescence intensity at this shorter wavelength to monitor heterodimer accumulation when HisH was combined with sub-stoichiometric HisF. Under conditions where the HisF-HisH heterodimer is in equilibrium with the free states of these enzymes, when [HisH] > [HisF], we deduced a linear function connecting [HisF-HisH] to [HisF], in which the slope depends on the heterodimer dissociation constant (*K*_d_). Based on this equation, taking fluorescence intensities as proxies of the heterodimer and HisF concentrations, we experimentally determined the *K*_d_ at four different temperatures. These *K*_d_ values were compared to those evaluated using ITC. Both methods revealed an increase in the HisF and HisH binding affinity as the temperature increases. In spite of differences in their absolute values, the *K*_d_ determined using these methods presented an evident linear correlation. To demonstrate the effectiveness of the fluorescence method we determined the effect on the *K*_d_ caused by 12 single mutations in HisF. Coherently, this test singled out the only mutation in the binding interface. In brief, the method described here effectively probes qualitative effects on the *K*_d_, can be carried out using common laboratory equipment and is scalable.

## Introduction

The HisF-HisH heterodimer, also known as Imidazole Glycerol Phosphate Synthase (IGPS) complex, is part of an important pathway that produces histidine and purines in bacteria, archaea, lower eukaryotes and plants [1, 2]. The bacterial HisF-HisH heterodimer is composed of two enzymes, a glutaminase, HisH, and a cyclase, HisF. The chain of reactions that this heterodimer catalyzes is initiated with HisH hydrolyzing glutamine to produce glutamate and NH_3_. Then, HisF incorporates that NH_3_ into its substrate, the nucleotide N’-[(5’-phosphoribulosyl) formimino]-5-aminoimidazole-4-carboxamide-ribonucleotide (PRFAR), producing imidazole glycerol phosphate (IGP) and 5-aminoimidazole-4-carboxamide (AICAR). IGP follows the route to histidine production, whereas AICAR is carried into the production of purines [3]. The participation of the HisF-HisH heterodimer in an important metabolic pathway in bacteria that is absent in humans and makes this heterodimer a potential target for antibiotic drugs [4]. For example, the inhibition of HisH by acivicin reduced *E*. *coli* growth in minimal media [5].

HisF and HisH possess a (β/α)_8_ barrel and a β/α hydrolase fold, respectively. In the heterodimer, HisH, which binds to the N-terminal face of the HisF barrel, presents the active site near the dimerization interface. However, the HisF active site is on the opposite face of the barrel, *i*.*e*., in the C-terminal face [6] (S1 Fig). As the activity of HisF depends on NH_3_ produced by HisH, the NH_3_ travels through a tunnel about 25 Å in length, which passes through the barrel-like structure of HisF, connecting the two active sites of these enzymes [3, 6–8].

In addition, the HisF-HisH heterodimer is an example of a V-type allosteric enzyme [3, 9], in which the allosteric mechanism has been described in detail [10, 11]. So, the binding of the PRFAR substrate to HisF active site produces an allosteric signal that increases the *k*_cat_ of HisH, speeding up the NH_3_ production. This activation involves modifications in the dynamics of the heterodimer, specifically in the a “breathing motion” in which the relative positioning of HisH and HisF oscilates around the “hinge” formed by the interacting residues fR249 and hW123 (f and h stands for HisF and HisH, respectively). Furthermore, small molecules that bind to the HisF-HisH heterodimer interface and affect the allosteric communication between these enzymes have been identified [9–17].

Therefore, the HisF-HisH interface is involved in multiple tasks: it keeps the heterodimer stably bound, it mediates the transfer of NH_3_ between active sites and it transduces the allosteric signal between them.

The analysis of the HisF-HisH heterodimer from the bacteria *Thermatoga maritima* (PDB 1GPW) reveals a dimerization interface covering about 1110 Å^2^ and involving 64 residues, 37 from HisF and 27 from HisH [7]. Sixteen residue pairs form hydrogen bonds, which are homogeneously distributed along the interface. In addition, three residue pairs are involved in salt bridges (S1 and S2 Tables; S2 Fig). Residues interacting in the interface tend to be conserved [3, 6, 7]. Moreover, analysis using ConSurf of 1,000 sequences of HisF and HisH from bacteria revealed that interface residues have variation on the conservation score, but 60% of them present a score higher than 7, from which 19 belong to HisH and 20 to HisF (S1 Table).

Therefore, the HisF-HisH heterodimer is an important model in the study of the biochemical and biophysical aspects of the protein-protein interactions. Its potential as a target for antibacterial drugs prompted us to develop a method for rapid analysis of the HisF-HisH heterodimer stability under different conditions.

## Material and methods

### Sequence alignments

Sequences of the HisF (UniProt Q9X0C6) and HisH (UniProt Q9X0C8) proteins were used as input for protein-protein BLASTp [18] in the non-redundant protein sequences database, searching only for bacteria sequences (taxid:2). The first 1,000 sequences were further analyzed. Sequence alignments and visualization were done using Clustal [19] web service and Jalview software [20]. Aligned sequences were used as input in ConSurf [21] to calculate conservation scores.

### Plasmids construction

The pET28a plasmids containing as insert the sequences coding for HisH and HisF from *Thermotoga maritima* were acquired from Genscript based on the sequence from UniProt entries Q9X0C8 and Q9X0C6, respectively. The insert coding for HisH was cloned into the plasmid using the restriction enzymes *Nde*I and *Xho*I, whereas the *Nde*I and *Eco*RI cleavage sites were used for HisF cloning. Both cloning strategies placed the inserts in frame to code for recombinant proteins that have an N-terminal His-Tag.

### HisF and HisH recombinant expression and purification

A single colony of bacteria transformed with the pET28a vector coding for HisF or HisH was selected and inoculated into two different vials with 5 ml of LB medium containing 50 μg/ml kanamycin. These samples were then cultivated for 16 h at 37°C. Then, they were separately inoculated into 500 mL LB containing 50 μg/ml kanamycin and incubated with shaking at 37°C, 200 rpm until the growth obtained an OD_600nm_ of 0.9. Next, 0.5 mM IPTG was added to induce the expression of the recombinant HisF or HisH, and a new round of incubation of 16 h at 30°C was performed. Finally, the bacterial cells were pelleted at 6,000 rpm for 30 min. Pellets were separately suspended in lysis buffer (10 mM sodium phosphate pH 7, 100 mM NaCl, 20 mM imidazole). Then, they were sonicated in a Branson 450 sonifier (5 pulses of 15 s at output 3, with intervals of 5 min on ice). After that, samples were incubated at 70°C for 20 min and then centrifuged at 16,000 rpm for 1 h. The supernatant was used for the recombinant proteins purification using the affinity of their His-tag to the Ni-NTA agarose resin (Qiagen). For that, each 1.5 mL of the supernatant was added to 0.25 mL of the resin. Then this mix was incubated for 1 h at 4°C with gentle shaking. Following that the resin was pelleted by centrifugation (13,200 rpm, 4°C for 5 min) and the supernatant was discharged. After 5 cycles of this washing procedure using the lysis buffer, the purified recombinant proteins were eluted from the pellet with 500 mM imidazole prepared in the same buffer. The recombinant protein purity was confirmed by SDS-PAGE [22]. Protein samples were submitted to buffer exchange using desalting columns PD mini-trap G-10 (GE Healthcare) following manufacturer instructions. The determination of soluble protein concentration was done using the molar extinction coefficients [23].

### Determination of changes in the dissociation constant (*K*_d_) for the HisF-HisH heterodimer using a method based on the intrinsic protein fluorescence

**Fig 1:**
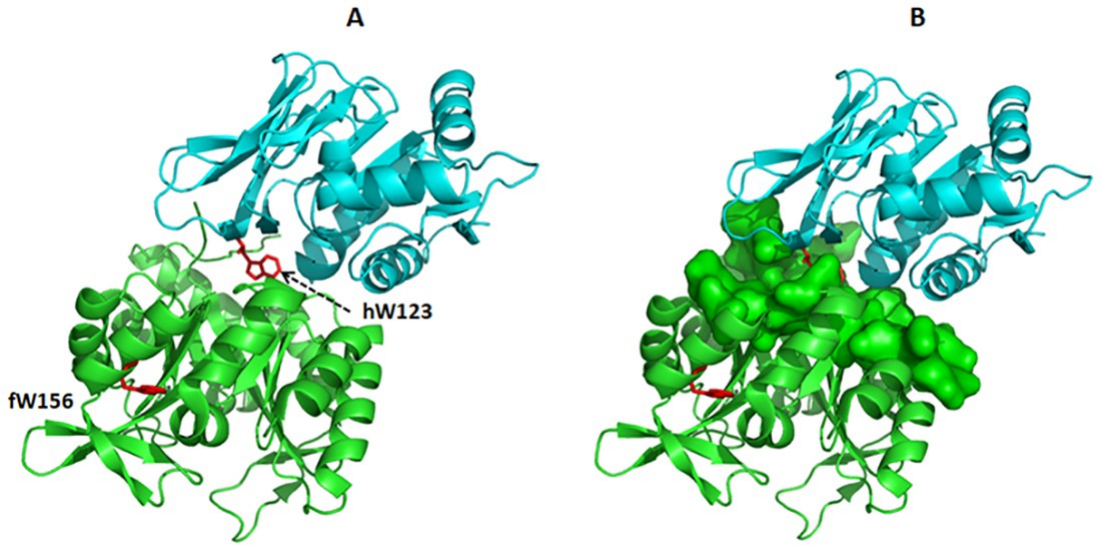
Interface of the HisF-HisH heterodimer showing the buried residue hW123. HisH is shown in cyan and HisF in green. A) Structure of the HisF-HisH heterodimer (PDB 1GPW) evidencing a single tryptophan residue (hW123 and fW156; in red) in both proteins. B) HisH has an exposed tryptophan residue (hW123) that is buried in the molecular surface of HisF residues in the heterodimer interface (green). Structures were visualized using Pymol Viewer.

The method for determination of changes in the affinity between HisH and HisF was based on the presence of only one tryptophan (hW123 and fW156) in both proteins, which have different maximum fluorescence wavelength when they are free or in the heterodimer state [3, 24]. Indeed, hW123 from HisH, which is placed in the heterodimer interface [7], is buried from the solvent upon dimerization (Fig 1). Thus, the intrinsic fluorescence at shorter wavelength (321 nm) was employed to detect HisF-HisH heterodimer formation.

A fluorescence emission spectrum was also collected for the isolated 15 μM HisH prepared in 20 mM potassium phosphate buffer pH 7.5. Next the fluorescence emission spectra of HisF were collected at different protein concentrations up to 5 μM. HisF was also prepared in 20 mM potassium phosphate buffer pH 7.5. In a separate experiment, fluorescence spectra were also collected for those different concentrations HisF samples in the presence of 15 μM HisH. For each HisF concentration, three fluorescence spectra (excitation at 295 nm and emission ranging from 310 to 400 nm) were acquired and averaged. The scan speed was 240 nm/min, and the slit opening was 2.5 nm for both excitation and emission. Experiments were conducted using a Fluorescence Spectrophotomer F7000 (Hitachi). The sample temperature was kept constant during the spectral acquisition by using a water circulation device connected to thermal bath (Multitemp III; GE Healthcare).

Next, we calculated the difference between the spectra of the samples containing simultaneously HisF and HisH (in which the heterodimer is formed) and the simple arithmetical sum of the spectra of the isolated HisH and HisF samples (which represent the hypothetical simple combination of HisF and HisH in the same media in the absence of interaction). This procedure was performed for each HisF concentration. From these difference spectra, we selected the data at 321 nm to describe the HisF-HisH heterodimer formation when HisF and HisH are combined and reach equilibrium. Next, those data were plotted against the fluorescence intensity (also at 321 nm) of each isolated HisF concentration, which corresponds to the initial HisF concentration when combined with HisH. It should be noted that the fluorescence intensities were used as a proxies for the protein concentrations.

A fit to the data presented in this plot was used to determine the *K*_d_ of the heterodimer based on the rationale described below.

The binding between HisF and HisH is described by a simple equilibrium

$$\begin{array}{lll} \text{HisF}+\text{HisH} & ⇄ & \text{HisF}-\text{HisH} \end{array}$$

The dissociation constant of the heterodimer is expressed as

$$K_{d}=\frac{\left[F\right][H]}{\left[FH\right]}$$

in which F stands for HisF, H corresponds to HisH and FH is the heterodimer HisF-HisH. Those are concentrations in the equilibrium.

Assuming a condition in which the initial [HisH] is higher than initial [HisF], *i*.*e*. [H_0_] > [F_0_], the formation of the heterodimer does not significantly alter [H_0_]. Hence [H] at any [F_0_] is similar to the initial concentration, *i*.*e*. [H] ≈ [H_0_]. On the other hand, the formation of the heterodimer changes [F_0_]. So [F] = [F_0_]–[FH]. Therefore, the dissociation constant is expressed as:

$$K_{d}=\frac{\left(\left[F_{0}\right]-\left[FH\right]\right)[H_{0}]}{\left[FH\right]}$$

$$\left[FH\right]=\frac{\left(\left[F_{0}\right]-\left[FH\right]\right)[H_{0}]}{K_{d}}$$

$$\left[FH\right]=\frac{\left(\left[F_{0}\right][H_{0}]-\left[FH\right][H_{0}]\right)}{K_{d}}$$

$$\left[FH\right]+\frac{\left[FH\right][H_{0}]}{K_{d}}=\frac{\left[F_{0}\right][H_{0}]}{K_{d}}$$

$$\left[FH\right](1+\frac{\left[H_{0}\right]}{K_{d}})=\frac{\left[F_{0}\right][H_{0}]}{K_{d}}$$

$$\left[FH\right]=\frac{\frac{\left[F_{0}\right][H_{0}]}{K_{d}}}{\left(1+\frac{\left[H_{0}\right]}{K_{d}}\right)}$$

$$\left[FH\right]=\frac{\left[F_{0}\right][H_{0}]}{K_{d}(1+\frac{\left[H_{0}\right]}{K_{d}})}$$

$$\left[FH\right]=\frac{\left[F_{0}\right][H_{0}]}{K_{d}+{[H}_{0}]}$$

$$\left[FH\right]=[F_{0}]\frac{\left[H_{0}\right]}{K_{d}+{[H}_{0}]}$$

Thus, the concentration of the heterodimer HisF-HisH in the equilibrium ([FH]) is a linear function of the initial concentration of HisF ([F_0_]), in which the intercept equals 0 and a slope can be used to calculate the *K*_d_ when the initial concentration of HisH ([H_0_]) is constant and known.

### Isothermal titration calorimetry

Isothermal titration calorimetry (ITC) was performed using a Nano ITC equipment (TA Instruments). The cell (170 μL) was filled with 58 μM HisH in 20 mM phosphate buffer, pH 7.5 containing 200 mM NaCl, identical to that used in the fluorescence experiments. A 50 μL syringe was filled with 400 μM HisF in the same buffer. Binding reactions were analyzed at 30, 40, 50 and 60°C. Injections of 2.5 μL of the HisF sample were used for the 30°C run, whereas injections of 2 μL were employed for the experiments at higher temperatures. The time interval between injections was 180 s. Stirring speed was kept at 150 rpm. All samples were degassed at each temperature before being inserted in the equipment. Reaction profiles and thermodynamic parameters were analyzed using the software NanoAnalyze v3.11.0 (TA instruments).

## Results and discussion

**Fig 2:**
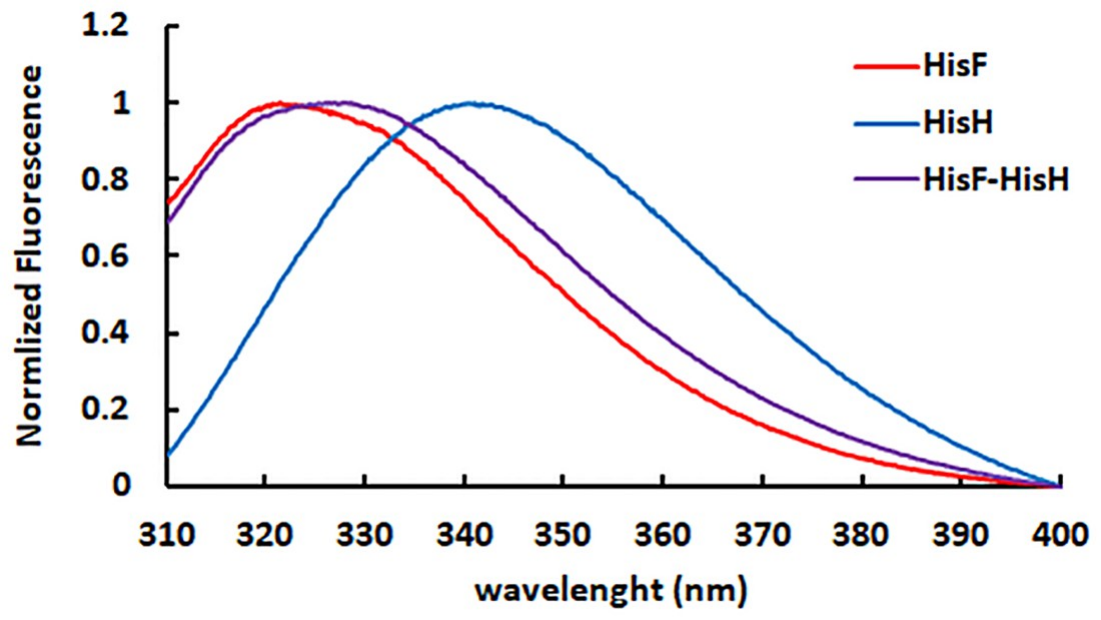
Effect of the dimerization on the HisF and HisH intrinsic fluorescence. The normalized spectra of HisF (red), HisH (blue) and HisF-HisH heterodimer (purple) present maximum fluorescence intensities at 321, 341 and 326 nm, respectively.

Isolated HisF and HisH show intrinsic fluorescence maxima at different wavelengths (321 and 339 nm, respectively), which reflects differences in the exposure to solvent of their single tryptophan residues (fW156 and hW123, respectively). Interestingly, the heterodimer HisF-HisH exhibits a fluorescence maximum at 326 nm, an indication that in this protein complex both tryptophan residues are buried (Fig 2), which is in agreement with previous observations [3, 24]. Indeed, hW123 from HisH, which is located in the heterodimer interface [7], is buried from the solvent upon dimerization (Fig 1). As previously commented, the shift of the intrinsic fluorescence maximum to a shorter wavelength (321 nm) is an indication of the HisF-HisH heterodimer formation [3, 24], hence we hypothesized that it could be employed to evaluate the affinity between HisF and HIsH.

**Fig 3:**
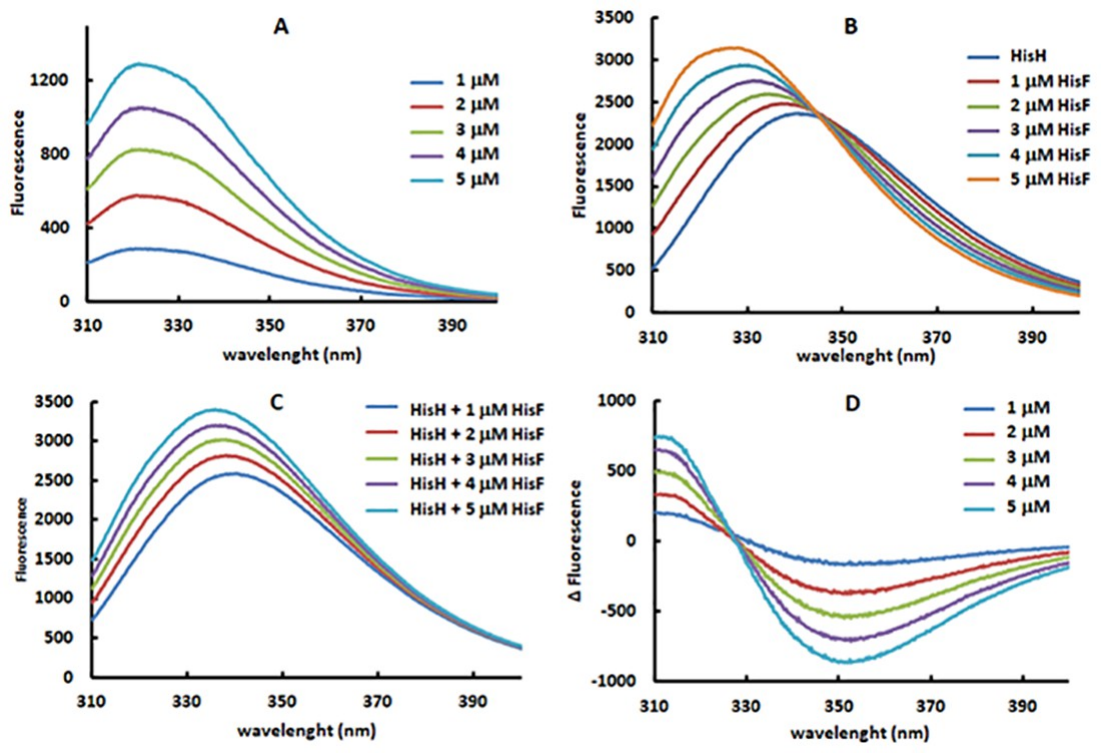
Intrinsic fluorescence spectra used in the determination of the dissociation constant of the HisF-HisH heterodimer. A) Fluorescence spectra of different concentrations of HisF. B) Fluorescence spectra collected along the mixing of 15 μM HisH with sub-stoichiometric HisF concentrations (1 to 5 μM). C) Arithmetical addition spectra combining the fluorescence of HisH (from B) with the fluorescence of different HisF concentrations (from A). D) Difference spectra calculated based on the arithmetical subtraction of the “addition spectra” (C) from the “mixing spectra” (B).

Firstly, we collected fluorescence spectra of HisF at various concentrations, ranging from 1 to 5 μM, and a spectrum of 15 μM HisH, higher than the HisF concentrations (Fig 3A). Subsequently we collected the spectra corresponding to the combination of 15 μM HisH with the sub-stoichometric HisF concentrations (Fig 3B). Those spectra contain the contribution of the different concentrations of the HisF-HisH heterodimer formed during this mixture. Next, we performed an arithmetical addition of the spectra of isolated HisH and HisF at different protein concentrations (Fig 3C). These calculated spectra represent the hypothetical simple combination of HisF and HisH in the same media in the absence of interaction. Hence, the difference between these two sets of spectra, the ones derived from the mixing experiment and those resulting from the arithmetical sum (Fig 3B and 3C), should reveal the fluorescence exclusively resulting from the HisF-HisH heterodimer formed along the mixing experiment (Fig 3D). Indeed, the positive differences calculated in the 310–330 nm range likely corresponds to the burial of the hW123 within the interface as the HisF-HisH heterodimer is formed.

Based on a simple equilibrium describing the HisF and HisH binding in a condition in which [HisH] > [HisF], we deduced an equation and the corresponding plot to calculate the dissociation constant (*K*_d_) of the HisF-HisH heterodimer (see Material and methods for a detailed description). In short, under these conditions the equilibrium concentration of the HisF-HisH heterodimer increases linearly with the initial [HisF] and the slope is inversely proportional to the *K*_d_. Such linearity should hold as long as the [HisF] does not approach [HisH]. Anyhow, this prerequisite is easily checked, as a linear plot is concrete evidence that the experiment obeys this restriction. It could be suggested that a very high [HisH] would assure that the [HisH] > [HisF] condition is obeyed along the experiment. However, it should be noted that as the line slope is equal to [HisH]/(*K*_d_ + [HisH]), the utilization of a very high [HisH] could result in [HisH] >> *K*_d_, then the slope would approach 1, *i*.*e*. it would be independent of the *K*_d_. Therefore, the initial HisH concentration has to be kept between these limits, *i*.*e*., it has to be higher than initial [HisF] and it should not excessively exceed the *K*_d_. Finally, we also observed that the intrinsic fluorescence can be used as a proxy of the HisF and HisF-HisH heterodimers concentrations, particularly the formation of the heterodimer is connected to the fluorescence in the 310–330 nm range (Fig 3D).

Holinski *et al*. had previously determined the *K*_d_ of bacterial HisF-HisH heterodimers employing a procedure based on the HisH intrinsic fluorescence [24]. It should be note that their procedure considers the analysis of stoichiometric combinations of HisF and HisH, which diverges from the conditions described here. Hence, those using these methods should be aware of their particularities.

**Fig 4:**
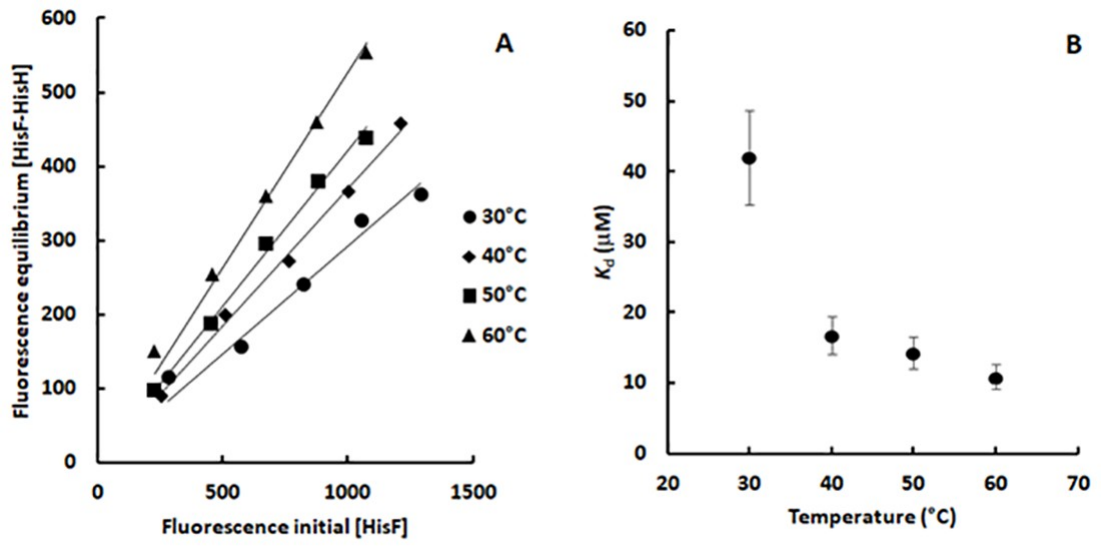
Determination of the dissociation constant of the HisF-HisH heterodimer at different temperatures. A) Effect of the initial HisF concentration ([*F*_0_]) on the HisF-HisH heterodimer concentration in the equilibrium ([*F*H]). Fluorescence readings at 321 nm were used as proxy for the HisF and the heterodimer concentration. Those data were extracted from Fig 3A and 3C, respectively. The linear correlation coefficients (*R*^2^) are 0.96, 0.99, 0.99 and 0.98 at 30°C, 40°C, 50°C and 60°C, respectively. The slopes are 0.29, 0.37, 0.42 and 0.53 at 30°C, 40°C, 50°C and 60°C, respectively. B) Effect of the temperature on the dissociation constant of the HisF-HisH heterodimer. The *K*_d_ were calculated based on the slopes of the lines shown in panel A. More details on Material and methods.

Then, we applied this method to estimate the *K*_d_ at four different temperatures (30 to 60°C) (Fig 4A). The plots show a good linearity, indicating that the approximations regarding the method and the experimental conditions were appropriate. Interestingly, the *K*_d_ data clearly shows that the HisF-HisH heterodimer affinity increases at higher temperatures, *i*.*e*., heterodimer formation is endothermic (Fig 4B). *T*. *maritima* can survive in an environment up to 90°C [25], so it is biologically plausible that at higher temperatures HisF and HisH have higher affinity.

**Fig 5:**
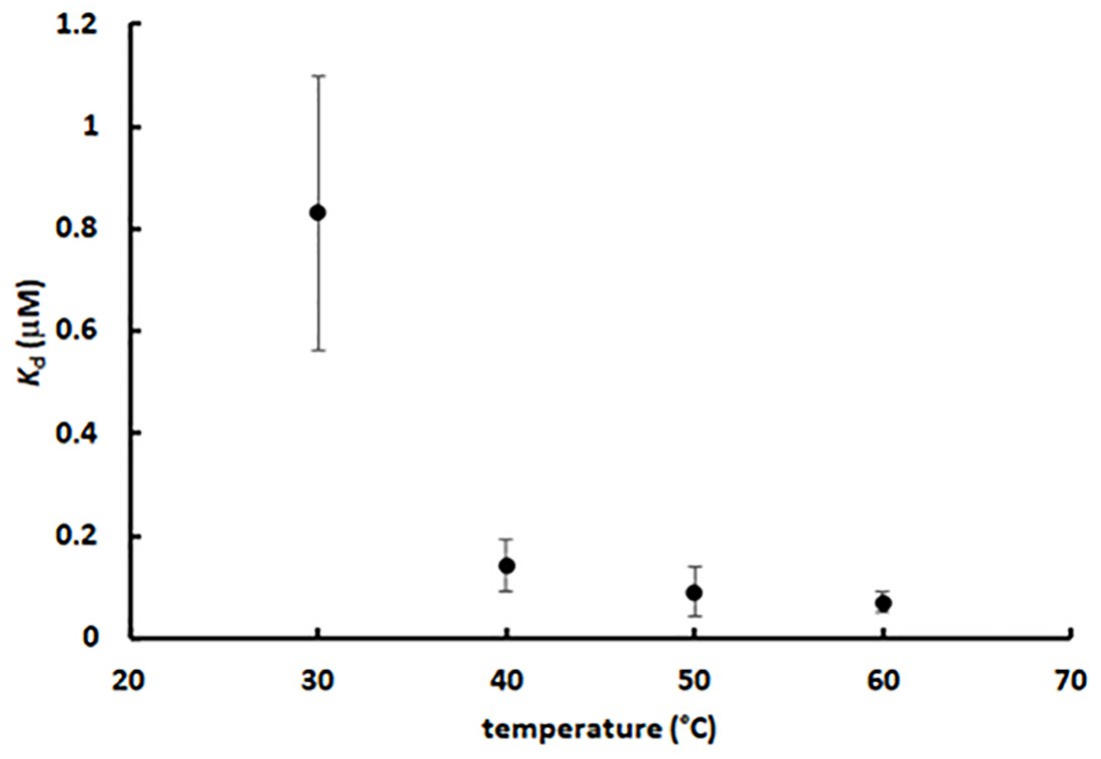
Determination of the dissociation constant of the HisF-HisH heterodimer at different temperatures using ITC.

We also probed the HisF-HisH heterodimer formation using ITC (Fig 5 and S3 Fig). This comparison is interesting to test our method for estimating *K*_d_ and also to gain deeper insight about the link between temperature and HisF-HisH affinity.

Experiments performed at 30°C showed higher noise/signal ratio in the heat rate (S4 Fig). Indeed, the binding enthalpy increases as the temperature gets higher and the heterodimer concentration increases. A larger deviation is also observed for the *K*_d_ determined at 30°C using the intrinsic fluorescence method (Fig 4). Thus, both experiments at 30°C seem to be revealing an intrinsic property of the HisF-HisH heterodimer, actually reaffirming the low affinity between HisF and HisH at this temperature. Hence, in spite of their larger uncertainties, they were included in the next analyses.

**Fig 6:**
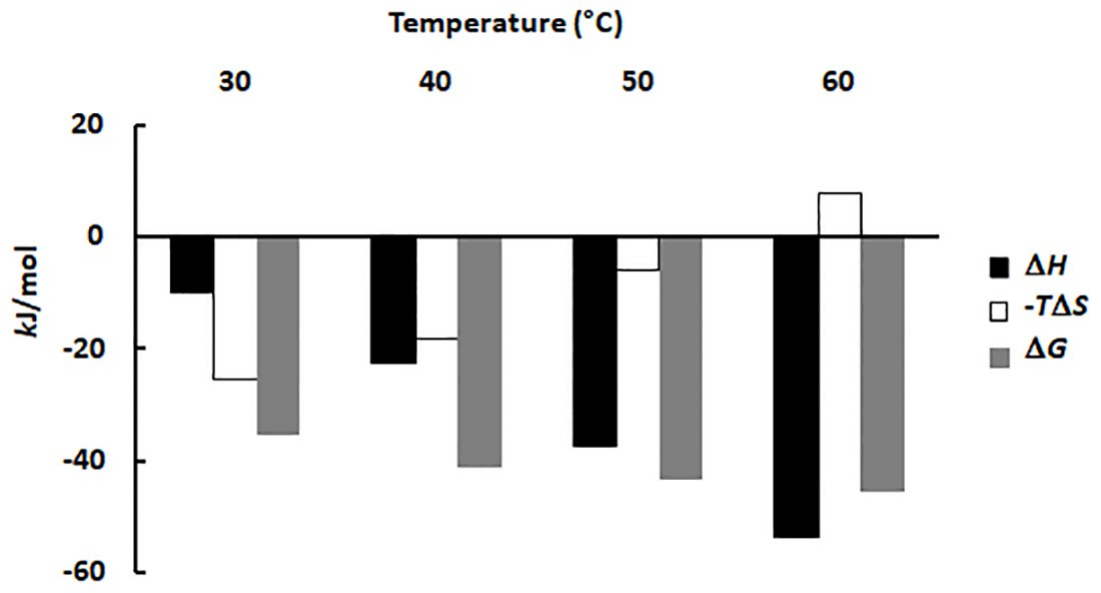
Thermodynamic parameters of the HisF and HisH binding calculated from the ITC experiments at different temperatures.

Interestingly, the HisF and HisH binding is entropically driven at 30°C, but that progressively changes as the temperature is increased. Actually, the enthalpy becomes the dominant binding factor above 40°C. Finally, at 60°C the entropy change is unfavorable, whereas the enthalpy change guides the binding (Fig 6 and S3 Table). Hence, at lower temperatures the interaction tends to be more dependent on the hydrophobic effect, whereas it relies on “polar contacts” (*i*.*e*., hydrogen bonds and ionic interactions) at higher temperatures. That is reasonable strategy for a heterodimer that should be stable in a large range of temperatures. Indeed, as the temperature increases the entropic gain resulting from the release of the solvation water from the monomers apolar surfaces should decrease because the molecules in the bulk solution having a high kinetic energy already display a large number of configurations (*i*.*e*., form disorganized system). Hence, a dimer relying only on the hydrophobic effect would become unstable as the temperature increases. In agreement, the HisF-HisH interface is rich in polar residues (75%) and 37% of them form hydrogen bonds or salt bridges (S1 and S2 Tables; S2 and S5 Figs).

**Fig 7:**
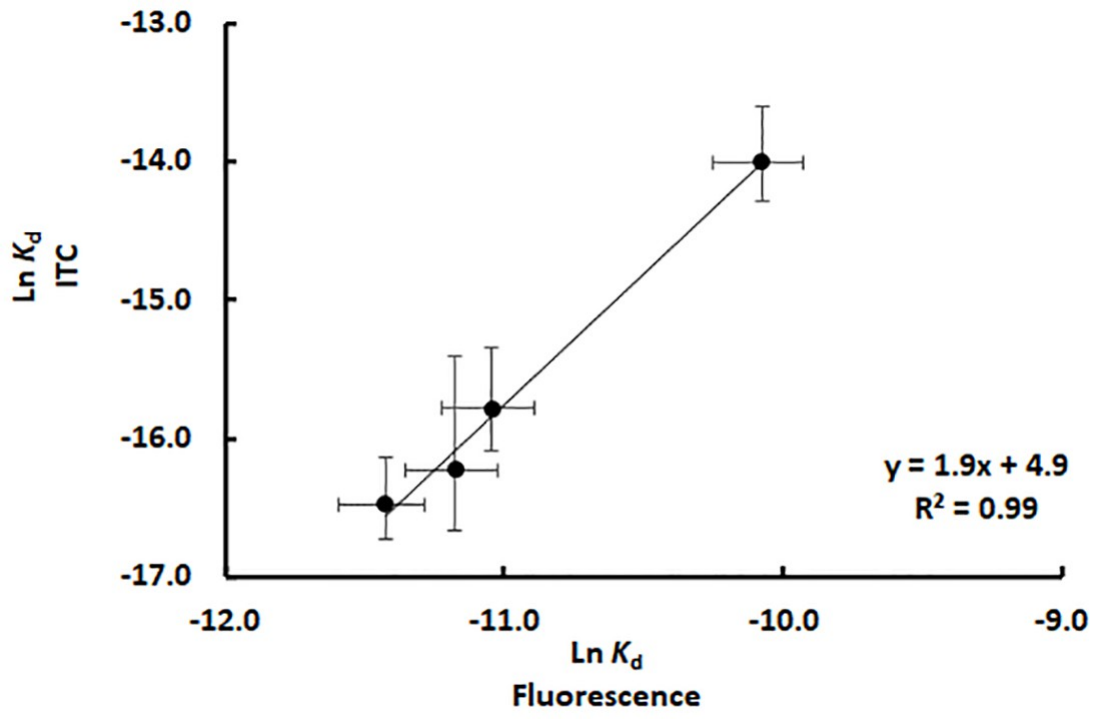
Correlation between *K*_d_ of the HisF-HisH heterodimer determined using the intrinsic fluorescence and ITC methods.

Notable, the heterodimer dissociation constant calculated using ITC and the intrinsic fluorescence method described exactly the same pattern showing that the HisF and HisH affinity increases as the temperature gets higher (Figs 4 and 5). That was also evidenced by evaluating the correlation between these dissociation constants (Fig 7). Note that as these constants show an exponential-like distribution with the temperature, when plotted in a linear scale the lower values tend to cluster, whereas the higher *K*_d_ value isolated. Therefore, the constants were converted to a logarithmic scale to produce a homogeneous distribution of the data points, improving the assessment of the linear correlation. As observed in Fig 7, *K*_d_ values determined using ITC and intrinsic fluorescence exhibited a good linear correlation, however, it should be noted that they diverged by two orders of magnitude. Such *K*_d_ divergence may arise from differences in the specific fluorescence of HisF and HisF-HisH heterodimer, *i*.*e*., intrinsic fluorescence intensity/mol of protein. So, the determination of this parameter and utilization of protein concentration in the plots like that in Fig 4 could minimize such divergence. Anyway, to make the procedure as simples as possible, we chose to maintain the intrinsic fluorescence as a proxy for the protein concentration.

Therefore, the fluorescence method presented here is a simple and quick method to probe qualitative effects on the *K*_d_, although it may not be an option for precise *K*_d_ determination.

**Fig 8:**
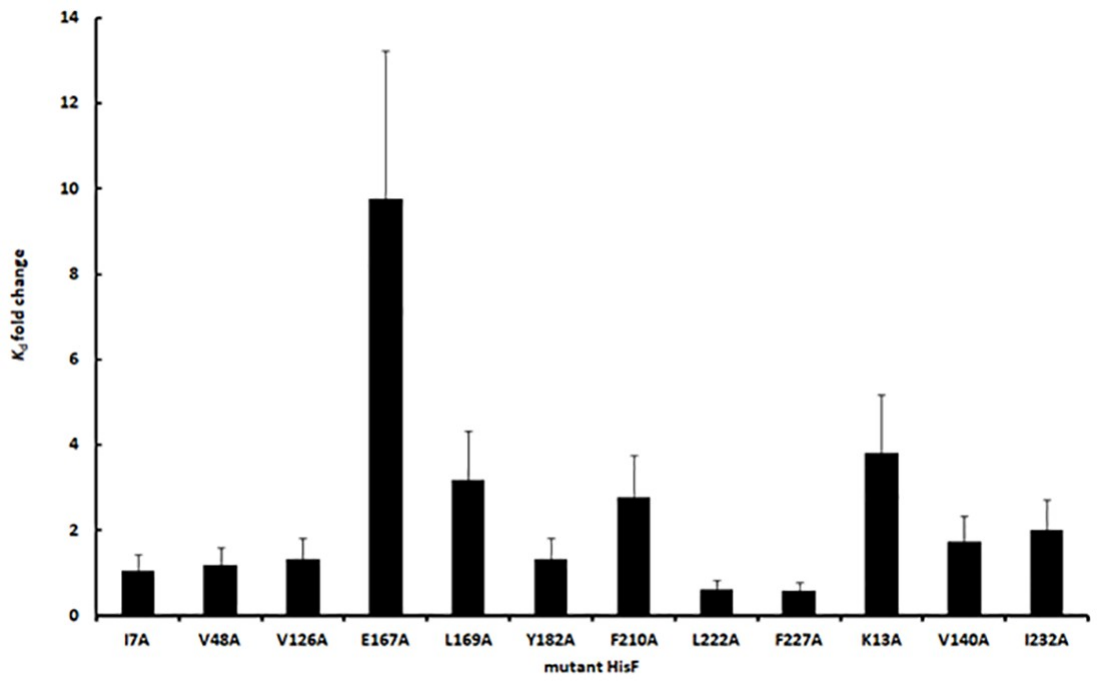
Effect of HisF mutations on the dissociation constant (*K*_d_) of the HisF-HisH heterodimer. Experiments were performed at 30°C. Error bars were based on the relative deviation of the dissociation constant in the experiments using the wild-type HisF (n = 6). Mutant HisF subunits are identified by the single residue exchange present on them.

As a demonstration of the utility of this method, we used it to determine the effect on the *K*_d_ of 12 single mutations in HisF (Fig 8).

This evaluation revealed that mutation E167A increased the *K*_d_ by 10-fold, the largest effect among the mutations analyzed here. Such clear affinity decrease is in agreement with the location of E167 in the heterodimer interface (S1 Table). Mutations K13A, L169A and F210A also increased the *K*_d_, but their effects were less substantial (about 3-fold). However, none of these residues is located in the heterodimer interface, suggesting they had an indirect effect. Remaining mutations, which also did not involve interface residues, produced no detectable effect on the HisF and HisH affinity. Thus, the fluorescence based method permit a swift screening of this mutant HisF set, coherently highlighting among them the mutant containing a replacement in the binding interface.

In conclusion the intrinsic fluorescence method described here is effective to probe qualitative effects on the *K*_d_ of the HisF-HisH heterodimer. In addition, it requires an equipment largely available (spectrofluorimeter) and it can be scaled up by using fluorescence plate readers. These characteristics make the intrinsic fluorescence method a good choice to screen the effect of mutations, small ligands and physical-chemical conditions on the HisF-HisH heterodimer formation and should be generally applicable to any oligomerization process that results in changes in fluorescence parameters.

## Supporting information

S1 Table Residues in the HisF-HisH heterodimer interface. (PDF)

S2 Table Residues in the HisF-HisH heterodimer interface that form hydrogen bonds and/or salt bridges between monomers. (PDF)

S3 Table Thermodynamic parameters of the HisF and HisH binding in different temperatures calculated from isothermal titration calorimetry experiments. (PDF)

S1 Fig HisF-HisH heterodimer showing the relative positioning between the active sites and the dimerization interface. (PDF)

S2 Fig Interface of the HisF-HisH heterodimer. (PDF)

S3 Fig Analysis of the HisF and HisH binding at different temperatures using isothermal titration calorimetry (ITC). (PDF)

S4 Fig Binding between HisF and HisH followed in isothermal titration calorimetry (ITC). (PDF)

S5 Fig Hydrophobicity index of the residues forming the interface of the HisF-HisH heterodimer. (PDF)
