# Supplementary material for: A simple method to determine changes in the affinity between HisF and HisH in the Imidazole Glycerol Phosphate Synthase heterodimer
Source: PLoS One. 2022 Apr 22;17(4):e0267536. doi: 10.1371/journal.pone.0267536 (PMC9032424; doi:10.1371/journal.pone.0267536)
Supplement: S1 Table — (PDF) [file pone.0267536.s001.pdf]

Supplementary Table 1 – Residues in the HisF-HisH heterodimer interface. Interface identification was performed in the PDBePISA server using the interface #2 of the structure 1GPW. Conservation score calculated in the ConSurf server. Score ranges from 1 to 9 (low to high conservation).

**HisF**		**HisH**	
Residue	Conservation score	Residue	Conservation score
MET 1	9	ASN 12	7
LEU 2	8	ASN 15	8
ALA 3	6	ARG 18	9
ARG 5	9	ARG 22	5
SER 40	4	ARG 117	1
GLU 41	1	PRO 119	8
ASP 45	9	HIS 120	7
GLU 46	9	MET 121	9
GLU 67	3	GLY 122	9
ALA 70	9	TRP 123	8
GLU 71	1	ASN 124	9
GLN 72	1	GLU 125	9
ILE 73	4	GLY 135	2
ASP 74	5	TYR 136	2
ILE 75	9	TYR 138	5
PRO 76	9	VAL 140	9
PHE 77	8	THR 142	9
ILE 93	9	GLU 157	4
LEU 94	1	TYR 158	7
ARG 95	6	ASP 159	2
GLY 96	9	GLU 180	9
ASP 98	9	LYS 181	9
LYS 99	9	SER 182	9
SER 122	9	SER 183	9
GLN 123	9	LYS 184	9
GLU 161	4	ILE 185	7
GLY 166	9	ARG 187	3
GLU 167	9		
THR 195	3		
LEU 196	7		
PRO 197	9		
ASN 247	1		
VAL 248	6		
ARG 249	9		
LEU 250	1		
GLU 251	1		
LEU 253	1		
