# Supplementary material for: A simple method to determine changes in the affinity between HisF and HisH in the Imidazole Glycerol Phosphate Synthase heterodimer
Source: PLoS One. 2022 Apr 22;17(4):e0267536. doi: 10.1371/journal.pone.0267536 (PMC9032424; doi:10.1371/journal.pone.0267536)
Supplement: S2 Table — (PDF) [file pone.0267536.s002.pdf]

Supplementary Table 2 - Residues in the HisF-HisH heterodimer interface that form hydrogen bonds and/or salt bridges between monomers. Interface identification and analysis was performed in the PDBePISA server using the interface #2 of the structure 1GPW. HB and SB refer to hydrogen bond and salt bridge, respectively. Conservation score calculated in the ConSurf server. Score ranges from 1 to 9 (low to high conservation).

**HisF**			**HisH**		
Residue	Interaction	Conservation Score	Residue	Interaction	Conservation Score
MET 1	HB/SB	9	ARG 18	HB	9
ALA 3	HB	6	ARG 22	HB	5
SER 40	HB	4	ARG 117	HB	1
GLU 41	HB	1	TRP 123	HB	8
ASP 45	HB	9	ASN 124	HB	9
ALA 70	HB	9	TYR 136	HB	2
GLU 71	HB	1	GLU 157	HB/SB	4
ILE 73	HB	4	LYS 181	HB/SB	9
ASP 74	HB	5	SER 182	HB	9
ILE 75	HB	9	SER 183	HB	9
ASP 98	HB/SB	9	LYS 184	HB	9
THR 195	HB	3			
ASN 247	HB	1			
