# Supplementary material for: A simple method to determine changes in the affinity between HisF and HisH in the Imidazole Glycerol Phosphate Synthase heterodimer
Source: PLoS One. 2022 Apr 22;17(4):e0267536. doi: 10.1371/journal.pone.0267536 (PMC9032424; doi:10.1371/journal.pone.0267536)
Supplement: S3 Table — (PDF) [file pone.0267536.s003.pdf]

Supplementary Table 3 - Thermodynamic parameters of the HisF and HisH binding in different temperatures calculated from Isothermal Titration Calorimetry experiments.

Temperature (°C)	ΔH (kJ/mol)	ΔS (J/mol.K)	$-T\Delta S$ (kJ/mol)	ΔG (kJ/mol)	n	K_d (μM)
30	-9.8±1	83.95	-25.45	-35.29	0.71±0.04	0.8±0.9
40	-22.7±0.4	58.69	-18.38	-41.14	1.02±0.01	0.14±0.05
50	-37.3±0.9	19.01	-6.142	-43.52	0.99±0.01	0.09±0.05
60	-53.6±0.8	-24.01	7.998	-45.69	1.04±0.008	0.07±0.02
