# Supplementary material for: A simple method to determine changes in the affinity between HisF and HisH in the Imidazole Glycerol Phosphate Synthase heterodimer
Source: PLoS One. 2022 Apr 22;17(4):e0267536. doi: 10.1371/journal.pone.0267536 (PMC9032424; doi:10.1371/journal.pone.0267536)
Supplement: S1 Fig — (PDF) [file pone.0267536.s004.pdf]

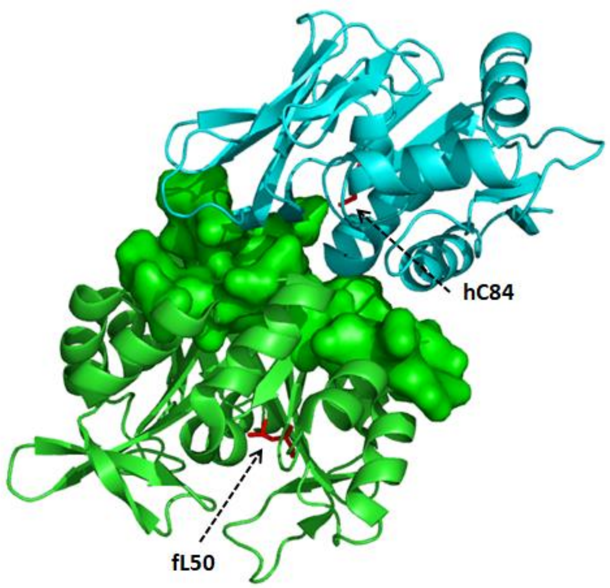

Supplementary Figure 1 – HisF-HisH heterodimer showing the relative positioning between the active sites and the dimerization interface. HisF is shown in green, whereas HisH is presented in cyan. Residues hC84 and fL50 located at the HisH and HisF active sites, respectively, are shown in red sticks. The HisF residues forming the dimerization interface are shown as a molecular surface in green. The HisH residues located at the dimerization interface are not shown to make easier the visualization of the HisH active site, which is close to that interface. For a representation of the HisH interface, see Supplementary Figure 2. Interface identification was performed in the PDBePISA server using the interface #2 of the structure 1GPW. Structures were visualized using Pymol Viewer.
