# Supplementary material for: A simple method to determine changes in the affinity between HisF and HisH in the Imidazole Glycerol Phosphate Synthase heterodimer
Source: PLoS One. 2022 Apr 22;17(4):e0267536. doi: 10.1371/journal.pone.0267536 (PMC9032424; doi:10.1371/journal.pone.0267536)
Supplement: S2 Fig — (PDF) [file pone.0267536.s005.pdf]

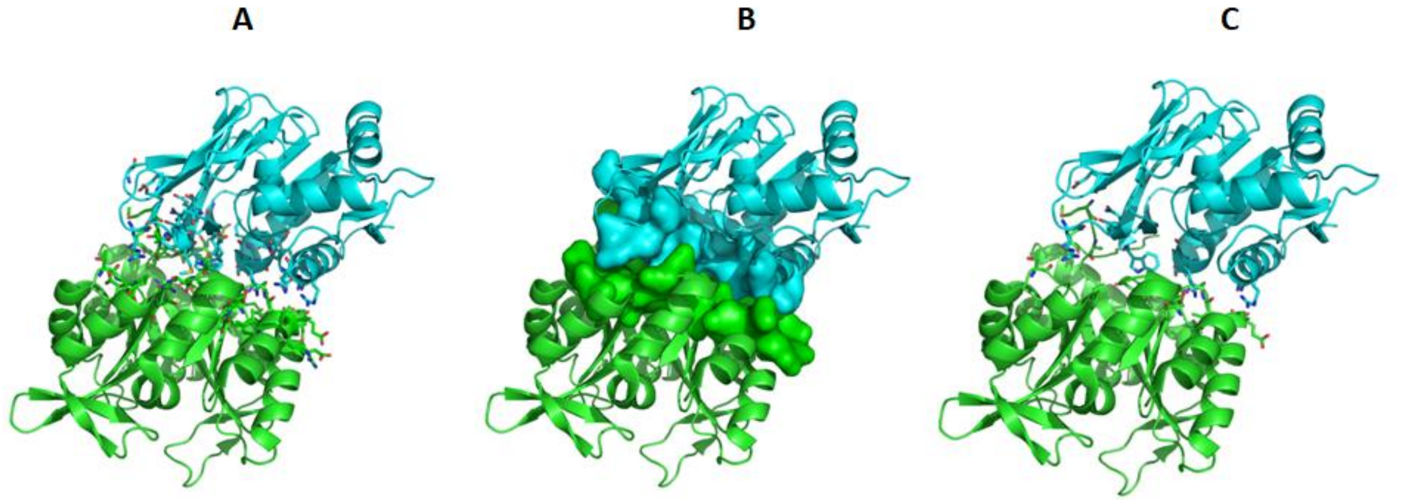

Supplementary Figure 2 – Interface of the HisF-HisH heterodimer. **A**) Residues forming the interface. HisF is shown in green, whereas HisH is presented in cyan. Residues in the heterodimer interface are rendered as sticks. **B**) Molecular surface of the residues forming the interface. **C**) Residues forming Hydrogen bonds in the interface. Interface identification was performed in the PDBePISA server using the interface #2 of the structure 1GPW. Structures were visualized using Pymol Viewer. Conservation scores for these residues, calculated in the ConSurf server, are presented on S1 and S2 Tables.
