# Supplementary material for: A simple method to determine changes in the affinity between HisF and HisH in the Imidazole Glycerol Phosphate Synthase heterodimer
Source: PLoS One. 2022 Apr 22;17(4):e0267536. doi: 10.1371/journal.pone.0267536 (PMC9032424; doi:10.1371/journal.pone.0267536)
Supplement: S3 Fig — (PDF) [file pone.0267536.s006.pdf]

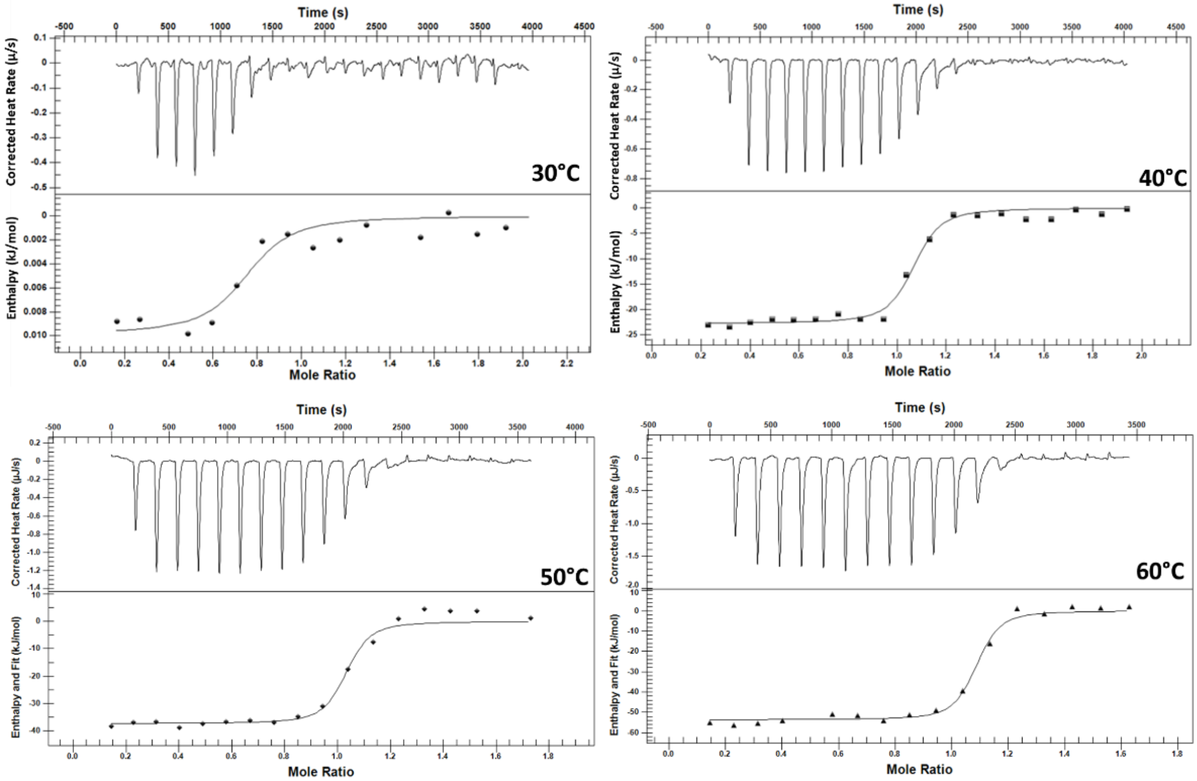

Supplementary Figure 3 – Analysis of the HisF and HisH binding at different temperatures using ITC. Corrected heat rate and integrated peaks enthalpy collected in the interaction of increasing concentration of HisF with 58 µM HisH.
