# Supplementary material for: A simple method to determine changes in the affinity between HisF and HisH in the Imidazole Glycerol Phosphate Synthase heterodimer
Source: PLoS One. 2022 Apr 22;17(4):e0267536. doi: 10.1371/journal.pone.0267536 (PMC9032424; doi:10.1371/journal.pone.0267536)
Supplement: S4 Fig — (PDF) [file pone.0267536.s007.pdf]

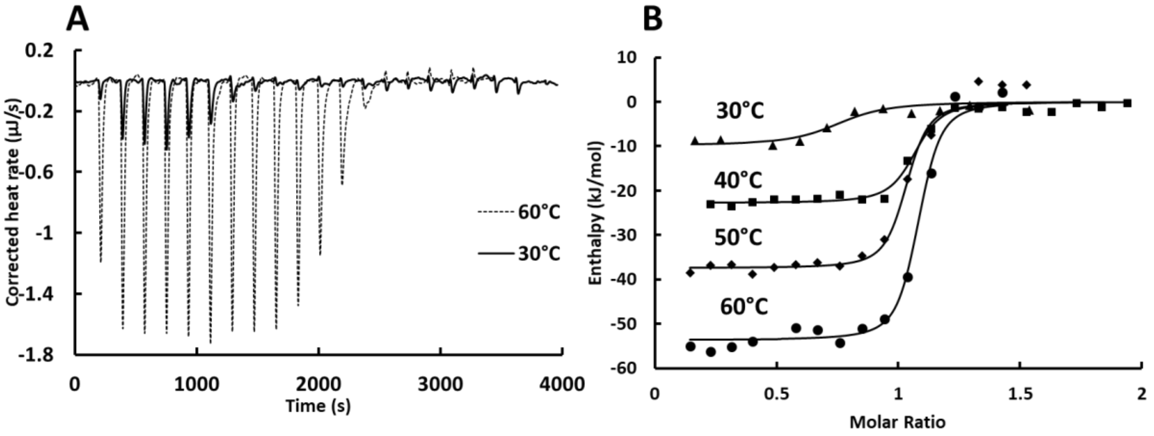

Supplementary Figure 4 - Binding between HisF and HisH followed in isothermal titration calorimetry (ITC). **A)** Corrected heat rate at 30 and 60°C showing the low signal generated by the binding reaction at 30°C when compared to a higher temperature. **B)** Integration of the peaks (enthalpy).
