# Supplementary material for: A simple method to determine changes in the affinity between HisF and HisH in the Imidazole Glycerol Phosphate Synthase heterodimer
Source: PLoS One. 2022 Apr 22;17(4):e0267536. doi: 10.1371/journal.pone.0267536 (PMC9032424; doi:10.1371/journal.pone.0267536)
Supplement: S5 Fig — (PDF) [file pone.0267536.s008.pdf]

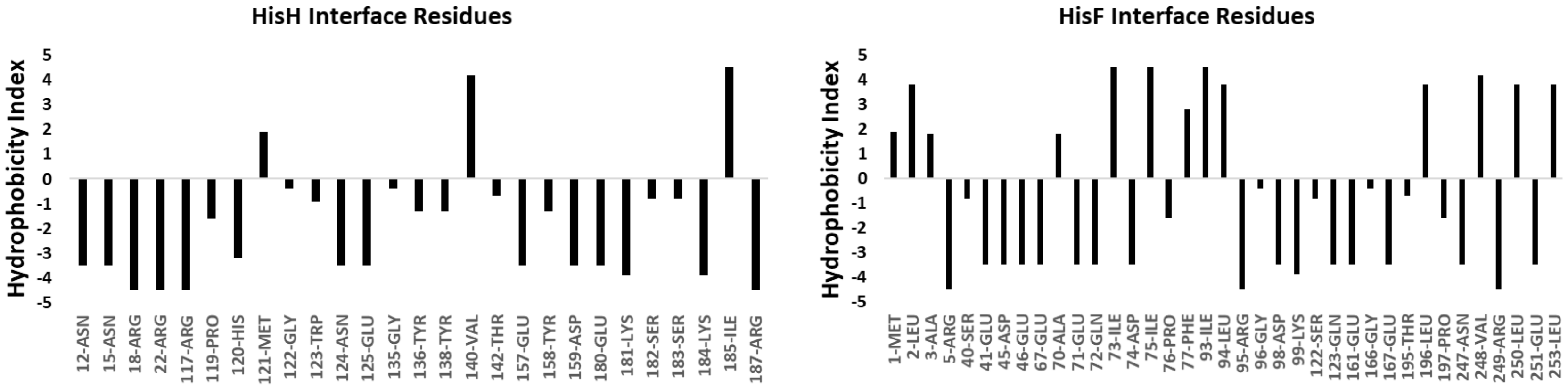

Supplementary Figure 5 - Hydrophobicity index of the residues forming the interface of the HisF-HisH heterodimer. Interface was identified using the chains C and D (interface #2) of the PDB1 GPW in PDBePISA server. Hydrophobicity index are accordingly to Doolittle and Kyte, 1982. Negative indices mean hydrophilic character.

Kyte J, Doolittle RF. A simple method for displaying the hydropathic character of a protein. J Mol Biol. 1982. doi:10.1016/0022-2836(82)90515-0
